# Spaceborne and UAV-LiDAR reveal hammer-headed bat preference for intermediate canopy height and diverse structure in a Central African rainforest

**DOI:** 10.1186/s40462-025-00552-7

**Published:** 2025-04-22

**Authors:** Nicholas J. Russo, Jean Michel Takuo, Valorian Tegebong, Matthew LeBreton, Morgan Dean, António Ferraz, Nicolas Barbier, Martin Wikelski, Elsa M. Ordway, Sassan Saatchi, Thomas B. Smith

**Affiliations:** 1 Department of Ecology and Evolutionary Biology, University of California, Los Angeles, CA USA; 2 Congo Basin Institute, Yaoundé, Cameroon; 3 Department of Animal Biology and Conservation, University of Buea, Buea, Cameroon; 4 Center for Tropical Research, Institute of the Environment and Sustainability, University of California, Los Angeles, CA USA; 5 Jet Propulsion Laboratory, California Institute of Technology, Pasadena, CA USA; 6 AMAP, Université de Montpellier, IRD, CNRS, INRAE, CIRAD, Montpellier, France; 7 Department of Migration and Immuno-Ecology, Max Planck Institute of Animal Behavior, Radolfzell, Germany; 8 Department of Biology, University of Konstanz, Constance, Germany

**Keywords:** GEDI, Frugivore, *Hypsignathus monstrosus*, Movement ecology, Pteropodidae, Step selection function, UAV-LiDAR, Vegetation structure

## Abstract

**Background:**

Animals with key ecological roles, such as seed-dispersing fruit bats, rely to varying degrees on habitat structure to indicate the locations of resources and risks.

**Methods:**

To understand how variation in vegetation structure influences fruit bat habitat selection, we related movement steps of hammer-headed bats (*Hypsignathus monstrosus*) to attributes of canopy height, vertical and horizontal vegetation structure, and habitat type in a mature rainforest of southern Cameroon. Vegetation structural metrics were measured with UAV-LiDAR at 10 m resolution for a 25 km^2^ study area. Because bats frequently moved outside the study area, we also characterized vegetation height and horizontal complexity over the full extent of bat movement trajectories by upscaling UAV-LiDAR measurements using primarily GEDI LiDAR data.

**Results:**

At the site level, hammer-headed bats preferred areas of intermediate canopy height (13.9–32.0 m) close to large canopy gaps (≥ 500 m^2^). Individual bats varied in selection for vertical vegetation complexity, distance to smaller canopy gaps (≥ 50 m^2^) and plant volume density of intermediate vegetation strata (10–20 m). Over the full extent of movement trajectories, hammer-headed bats consistently preferred intermediate canopy height, and areas closer to canopy gaps. At both spatial extents, bats moved the shortest distances in swamp habitats dominated by *Raphia* palms. These behaviors indicate the use of forest types that vary structurally, with a preference for open airspace during foraging or moving among resources, and for dense swamp vegetation during roosting and resting periods. In addition, most bats regularly made long flights of up to 17.7 km shortly after sunset and before sunrise and limited their movements to three or fewer destinations throughout the tracking period.

**Conclusions:**

These results highlight the importance of structurally diverse landscapes for the nightly movements of hammer-headed bats. Our results show how remote sensing methods and animal tracking data can be integrated to understand habitat selection and movement behavior in tropical ecosystems.

**Supplementary Information:**

The online version contains supplementary material available at 10.1186/s40462-025-00552-7.

## Background

Animal movements have critical consequences for ecosystem functioning and viral spillover, but are underexplored in tropical habitats. Fruit bats (Pteropodidae) are important long-distance seed dispersers [1] and viral reservoirs in tropical ecosystems [2]. For example, a single colony of straw-colored fruit bats (*Eidolon helvum*) in Ghana can disperse hundreds of thousands of seeds in one night, and up to 95 km—among the longest distances of any known disperser [1]. Beyond the economic value this ecosystem service provides for tropical reforestation, seed dispersal by bats ultimately influences the genetic diversity and species composition of rainforest tree communities [3, 4]. Understanding the movements of fruit bats is also potentially important in predicting disease transmission. Many bat species have an unusually high virus tolerance and may, consequently, act as viral reservoirs, although robust evidence for bats as reservoir hosts is lacking in most African study systems [5]. However, changes in bat behavior and resource selection—especially those that lead bats to come into contact with humans—are sometimes thought to heighten the possibility of viral spillover [2, 6].

Animals evaluate landscapes according to the distribution of resources and risk. Resources include foraging, resting, and nesting areas, while risks include predation and thermal stress [7]. Vegetation structure can indicate the locations of resources and influence route use [8, 9]. Three-dimensional vegetation structure has been shown to shape bat communities, with some species preferring denser vegetation and others preferring open airspace for foraging [10]. Vegetation can sometimes hinder maneuverability by obstructing bats’ flight paths [11]. Characterizing 3D vegetation structure at fine scales (sub-meter resolution) is possible with terrestrial, drone-mounted, and airborne Light Detection and Ranging (LiDAR) [12]. This capability enables ecologists to quantify the 3D space use of arboreal and aerial animals [13]. However, animals may move across seasonal home ranges that exceed extents that can be surveyed by high-resolution LiDAR. Spaceborne LiDAR, including the recent Global Ecosystem Dynamics Investigation (GEDI) mission, addresses this problem by collecting 3D vegetation structure data at a near-global extent [14], albeit with gaps in spatial coverage due to its sampling design. Spaceborne LiDAR is therefore a promising tool for understanding how animals evaluate landscapes throughout migrations, dispersal, and nomadic movements.

Remote sensing can provide information about animal habitats that would not be possible from collecting in situ data, but there are still challenges in characterizing landscapes in ways that are relevant to animal behavior [15]. Animals may prefer habitat features at different spatial and temporal scales [16, 17], and many remote sensing measurements—which are not normally designed for animal ecology—are imperfect or indirect indicators of preferred habitat features. Because habitat features are difficult to characterize at fine scales and over large spatial extents, recent methods have presented “upscaling” procedures that infer missing measurements based on machine learning [18]. Through this approach, maps of canopy height and structural complexity have become available at global scales [19–21]. Widespread adoption of machine learning methods to improve large-scale characterizations of animal habitat can greatly advance ecological research.

It is advisable for movement ecology research to keep pace with advances in remote sensing, which now enable the characterization of 3D vegetation structure at broad spatial extents [15]. Some species of fruit bats fly dozens of kilometers per night [1], encountering a variety of habitats as they commute among resources, and often using social cues to gather information [22, 23]. Well-studied species display advanced spatial memory of fruiting trees and roosting sites [24, 25]; in fact, the Egyptian fruit bat (*Rousettus aegyptiacus*) creates new routes among these resources using cognitive map-based navigation [25]. Disentangling the role of remotely-sensed landscape features in predicting bat movement behavior will help guide conservation decisions and predict disease spread [6]. Continued research linking animal movement to remotely sensed landscape features can address how animals move in relation to landscape features, and how their movements influence vegetation structure through seed dispersal and nutrient transport [26].

Hammer-headed bats (*Hypsignathus monstrosus*) are a lekking species that can be found in mature rainforests, rural settlements, and urban areas in Central and West Africa, and they are the largest fruit bat species of continental Africa [27, 28]. This species has been observed migrating along the Congo River, and movements up to 10 km have been tracked previously at a lek in the Republic of the Congo [27, 29]. Still, next to nothing is known about potential migratory movements of hammer-headed bats, which are a suspected—but unconfirmed—reservoir of *Ebolavirus* [30, 31]. A GPS tracking study revealed that hammer-headed bats prefer agricultural areas in a managed forest-agricultural landscape, and typically move along waterways [27]. Because hammer-headed bats often vocalize in large canopy gaps and roost in dense vegetation, we expected 3D vegetation structure to influence their habitat selection in a mature tropical lowland rainforest.

We aimed to reveal the attributes of 3D vegetation structure that influence hammer-headed bat movements. Specifically, we explored (1) Individual-level selection for 3D vegetation structure and habitat types at 10 m spatial resolution, (2) Population-level selection for vegetation structure at coarser spatial resolution (30 m) and across the full extent of bat movement trajectories, and (3) Nightly movement distances and recursions to locations of high use.

## Methods

### Study site

**Fig. 1:**
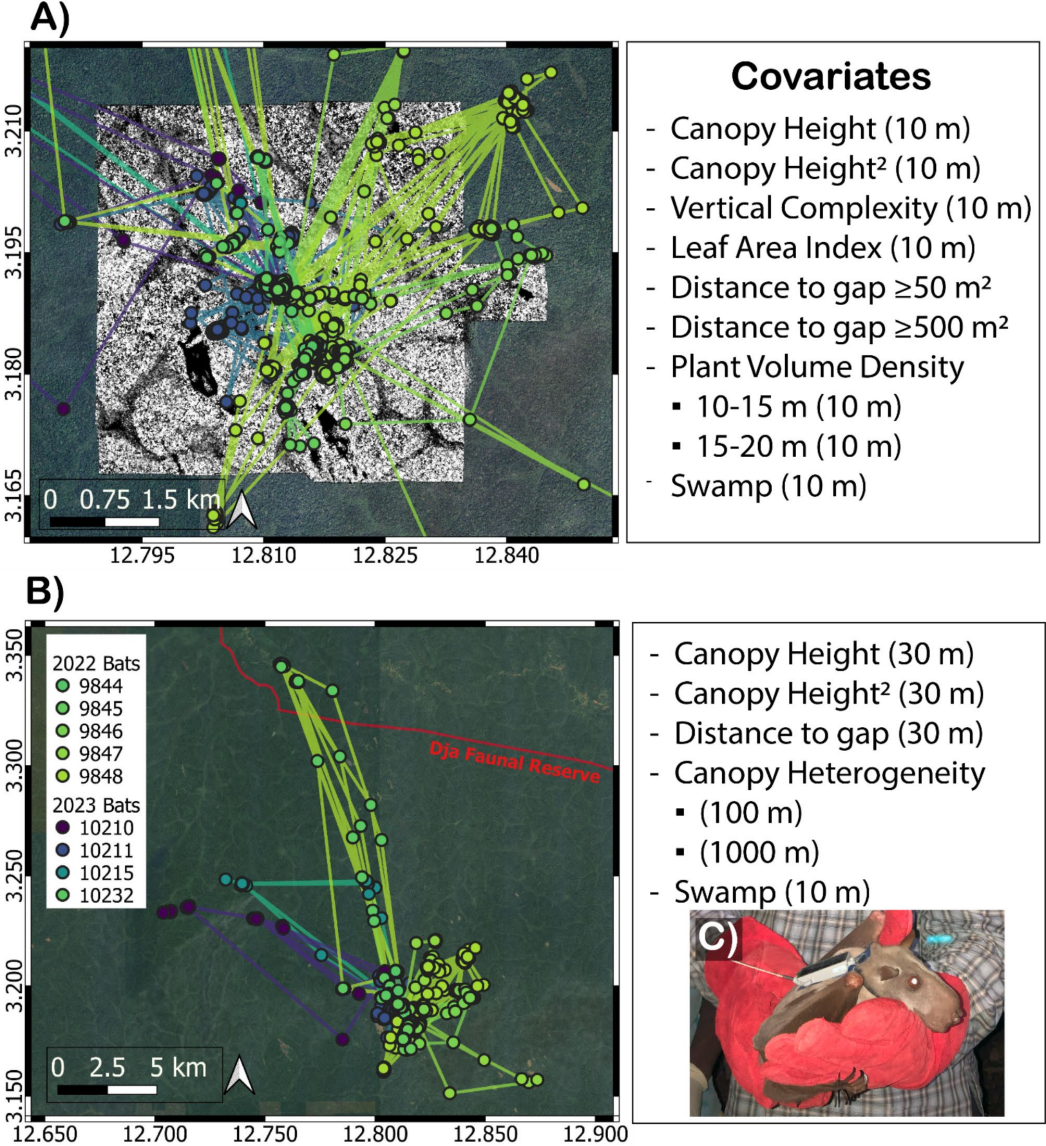
Movement trajectories of all bats and a depiction of the two spatial extents of habitat selection analyses. **(A)** Attributes of 3D vegetation structure measured at 10 m resolution were limited to the 25 km^2^ site level within the UAV-LiDAR extent of Bouamir Research Site. Bat movement tracks are overlain on a map of canopy height (black = 0 m, white = 55 m). Note that canopy height (height of first LiDAR Return) was included in all models as a quadratic term (canopy height + canopy height^2^). Vertical complexity: total diversity of 3D point cloud distribution measured from ground to top-of-canopy; Distance to gap: straight-line distance to nearest area with no vegetation > 5 m; Plant Volume Density: leaf area per volume within a specified height bin (10–15 or 15–20 m). Swamp: habitat characterized by seasonal or permanent shallow water and characterized by dominance of *Raphia* palm species. **(B)** We used upscaled 3D vegetation structure metrics to quantify habitat selection at the landscape level, which encompassed the full scale of bat movement tracks, including the boundary of the Dja Faunal Reserve. Canopy height: predicted value of 95th percentile relative height (RH 95). Distance to gap: straight-line distance to nearest area with no vegetation > 15 m. Canopy heterogeneity: standard deviation of canopy height at a specified spatial resolution (100–1000 m). Swamp: same as in panel A. **(C)** The inset photo shows a male hammer-headed bat carrying a 15 g e-obs tag

All field research took place within the Bouamir Research Site (hereafter, “Bouamir”), a 25 km^2^ study area near the center of the Dja Faunal Reserve in southern Cameroon (3°11’ N, 12°48’ E). The site comprises mainly *terra firma* forest, *Raphia* palm-dominated swamps, and grass-covered peaks called inselbergs. A LiDAR survey for the entire study site was completed with an unoccupied aerial vehicle (UAV-LiDAR) in March 2022, providing a 3D point cloud with an average density of 300 points · m^− 2^ (Reddy et al. 2024; Fig. 1A).

### Bat capture and tracking

We captured bats using mist nets (38 mm gauge) placed in the canopy in front of known roosts within Bouamir and captured five bats each in Aug. 2022 and Aug.-Sep. 2023 (n = 10 total bats). We constructed and operated canopy mist nets following [32] from sunset until sunrise. We tagged seven males and three females with a solar-powered 15 g GPS tag containing an accelerometer (manufactured by e-obs). Tags were glued to a lightweight “cape” fastened around the neck using a 0.9525 cm (3/8”) strap (BioThane) secured with a plastic snap rivet, similar to [29] but with different materials. Tags collected a GPS location every thirty minutes from 17:00–7:00 local time. We downloaded GPS data from each tag throughout the tracking period using an e-obs BaseStation with a 10-element Yagi antenna. We retrieved 3–15 nights of data from nine bats (Fig. S1) and used GPS data from these individuals for analyses. We did not retrieve enough data from the tenth bat. All capture and tracking methods were approved by Cameroon’s Ministry of Scientific Research and Innovation and Ministry of Wildlife and Protected Areas, and the University of California, Los Angeles Animal Research Committee, under protocol #2019-037-01.

### Habitat selection at site level (25 km^2^)

We quantified habitat selection of each bat based on seven structural metrics measured with UAV-LiDAR within the Bouamir Research Site, representing canopy height, vertical complexity, and canopy cover (Fig. 1A; descriptions in Table S1). After an initial period of data exploration, it appeared that most of the bats preferred areas with intermediate canopy height relative to available habitat, so we chose to include a quadratic term for canopy height that would capture a potential nonlinear relationship. Because the study site includes three major habitat types (*terra firma* forest, *Raphia* palm-dominated swamps, and inselbergs), we delimited these habitat types using a Convolutional Neural Network (CNN) applied to a composite, cloud-free Sentinel-2 image centered on Cameroon’s rainforest zone and covering 178,930 km² [33]. We implemented the CNN by identifying land cover type (e.g., forest, swamp, and inselberg) of 10,084 polygons within the Sentinel-2 image and inferring the distribution of each habitat type in the full image using Orfeo ToolBox [34], with 80% of polygons used for training and 20% for validation. We included two habitat categories in habitat selection analyses, “swamp” and “non-swamp”, because two bats rarely or never encountered inselbergs.

We quantified each individual bat’s selection for each habitat variable using an integrated Step Selection Analysis (iSSA), which uses a conditional logistic regression to estimate parameters of habitat selection behavior and movement behavior together in the same model. The iSSA compares bat movement “steps”—the straight-line distance between successive GPS locations—to 100 randomly generated steps based on the observed distribution of step lengths and turn angles [35]. We included the log-transformed step lengths and cosine of the turn angles as metrics of movement behavior in our models [36]. We added 1 m to all step lengths prior to log-transformation so that any sedentary periods would yield a value of zero or greater. We scaled and centered each continuous habitat covariate before inclusion in analyses and only included covariates in models that were not highly correlated (Pearson’s correlation coefficient <|0.6|). Field observations of bat roosting locations and suspected foraging locations led us to test the hypothesis that movement step lengths were shorter in swamp habitats, and so we also included a term representing the interaction between movement step length and use of swamp habitat in iSSA models. We determined the direction and magnitude of selection for each covariate based on selection coefficient estimates from the iSSA. All iSSA models were fit using the “fit_issf” function in the “amt” R package (version 0.2.1.0) [37].

To determine the influence of canopy height on habitat selection for each bat, we calculated the Relative Selection Strength (RSS) for each value of canopy height relative to the mean canopy height of habitat available to the bats, while holding all other covariates constant [38]. This metric enabled us to characterize a nonlinear relationship between canopy height and habitat selection for each bat. We assessed the fit of iSSA models to each individual bat with used habitat calibration (UHC) plots, which compare predicted values of each covariate to the distributions of both selected and available habitat using *k*-fold cross validation [39]. We simulated 1000 distributions of all covariates except the interaction term, and using k = 5 folds, using the “prep_uhc” function in the “amt” R package [37].

Finally, we used a generalized linear mixed effects model (GLMM) to estimate population-level selection for each covariate, using the “glmmTMB” R package (version 1.1.7) [40, 41]. This model included all covariates from the iSSAs as fixed effects and treated individual bat IDs as a random effect.

### Habitat selection at the landscape level

Because seven of the nine bats flew beyond the 25 km^2^ study area surveyed by UAV-LiDAR, we needed to characterize vegetation structure over the full extent of the bats’ movement trajectories to understand habitat selection at the landscape level. We hypothesized that canopy height influences bat habitat selection and therefore targeted this metric and three derivatives: location of canopy gaps and height heterogeneity at two different spatial resolutions (100 and 100 m; Fig. 1B). We used data from the GEDI spaceborne LiDAR because its measurements cover most of the world’s temperate and tropical regions at 25 m resolution. However, GEDI’s pervasive gaps in spatial coverage required us to interpolate measurements of vegetation structure using a Random Forest algorithm. The resulting product was a continuous-coverage (“wall-to-wall”) map of canopy height for a 300 km buffer around Cameroon’s Dja Faunal Reserve (~ 494,000 km^2^), which covered the full extent of bat movement trajectories. Characterizing vegetation structure at the landscape level required two main steps: (1) Calibrate GEDI measurements using airborne and UAV-LiDAR measurements and (2) Interpolate canopy height values where GEDI data are unavailable using optical and radar measurements.

Calibrating GEDI measurements required comparing airborne and UAV-LiDAR measurements to GEDI measurements in areas where they overlap—in the Congo Basin, we selected three drone LiDAR surveys in Cameroon [42] and 211 airborne LiDAR samples from the Democratic Republic of the Congo [43]. We used the vegetation relative height 95th percentile (RH 95) metric from GEDI Level 2 A (L2A) data as our target variable for creating a wall-to-wall map of canopy height [14, 44] because it best represents canopy height while filtering out potential anomalies. The RH 95 product represents a composite image of data collected from 2019 to 2022. As with many LiDAR studies, we assumed that vegetation structure does not differ significantly between the time of LiDAR acquisition and the time an animal visits the location, or that the location’s vegetation structure relative to the rest of the landscape will vary significantly through time [45]. We filtered the GEDI measurements to improve quality (e.g., eliminate cloud-covered pixels) and to find the closest comparison between GEDI measurements and reference airborne and UAV-LiDAR data. To calibrate the RH 95 measurements, we systematically tested combinations of GEDI filters through six different algorithm setting groups, each of which retrieves the location of the ground, with error propagating to the RH 95 estimate [46]. We identified the fifth GEDI quality algorithm as optimal, with the quality flag filter equal to 1 and sensitivity filter ranging from 0.98 to 1. To further ensure data quality, we manually removed erroneous GEDI shots that were not filtered out by this method but displayed unnatural patterning along orbital tracks that indicated them as outliers (Fig. S2).

Because none of the LiDAR methods provide data over the full extent of bat movements, we trained a Random Forest algorithm using a third set of remote sensing variables with continuous coverage in the region of interest. These variables, which included measures of vegetation reflectance (from Landsat 8) and radar backscatter values (from Sentinel-1 and ALOS/PALSAR) do not directly measure canopy height but vary according to both vegetation height and cover [47, 48]. In other words, we predicted canopy height in areas not covered by the GEDI scanner using optical and radar measurements, based on the relationship between these variables and canopy height. The Random Forest algorithm works by generating multiple decision trees trained from a random subset of data with input variables, where the final prediction—in our case, canopy height—is the unweighted average decision of the collection of trees [44]. We trained the Random Forest using 14 input variables that we expected to indicate or influence vegetation height, including Landsat 8 bands 2–7 and NIRv (near-infrared reflectance strictly from vegetation), Copernicus Digital Elevation Model (DEM), Copernicus DEM-derived slope and aspect, and Synthetic Aperture Radar measurements from ALOS PALSAR-2 (HH and HV), and Sentinel-1 (VV and VH). We generated a 30 m canopy height map for the region because, unlike available products, our analysis was fit to southern Cameroon and therefore more locally accurate than global or pan-tropical canopy height maps, and it provided complete coverage of the 494,000 km^2^ study area [19, 20].

We derived canopy height heterogeneity by aggregating canopy height values to 100 m and 1000 m resolution and calculating the standard deviation of the 30 m pixels within each grid cell. We also characterized canopy gaps at the landscape level using our 30 m canopy height map and the “getForestGaps” function from the “ForestGapR” R package [49]. Unlike at the site level, where canopy gaps were defined as pixels with no vegetation taller than 5 m [50], landscape-level canopy gaps were characterized as areas with no vegetation taller than 15 m. This definition increased the sensitivity of our methods to detect canopy gaps. We included an upper area threshold of 500 ha for canopy gaps to include large villages but avoid including river surface area as canopy gaps, which we consider functionally different as a landscape feature [27]. We generated a raster representing distance to nearest canopy gap in meters using the “distance” function in the “terra” R package (version 1.7–39) [51]. We included the terms for swamp and the interaction between swamp habitat selection and movement step length in the landscape-extent model. These were the only two terms included at the same resolution and extent in both the site- and landscape-extent models. We tested the effects of all landscape-level covariates (Fig. 1B) on population-level habitat selection using the same methods described in the section “Habitat selection at the site level (25 km^2^)” and further explored the difference in step lengths through swamp vs. non-swamp habitats using a Wilcoxon signed-rank test.

### Movement behavior

Fruit bats are known to repeatedly visit resources with directed movements [25, 27], known as “recursions” [52]. For each bat, we quantified the number of recursions to a 100 m radius around each GPS location using the “getRecursions” function in the “recurse” R package (version 1.1.2) [53]. We used a *k*-means clustering algorithm to identify up to three centroids of recursions throughout each bat’s movement trajectory, representing the 75th percentile of recursions or greater [54].

Because some of the bats appeared to commute to 1–3 locations after sunset and remain within a small radius at those locations, we were also interested in how step lengths varied with time since sunset. We explored this relationship using a generalized additive mixed model (GAMM) with a smoothed term for hours after sunset, implemented in the “mgcViz” R package (version 0.1.11) [55]. We also summarized both the distances between each bat’s successive GPS locations and distances from the capture location (Maximum Net Squared Displacement) using the “adehabitatLT” package (version 0.3.27) [56]. All analyses were conducted using R version 4.3.1 [57].

## Results

### Habitat selection at the site level (25 km^2^)

**Fig. 2:**
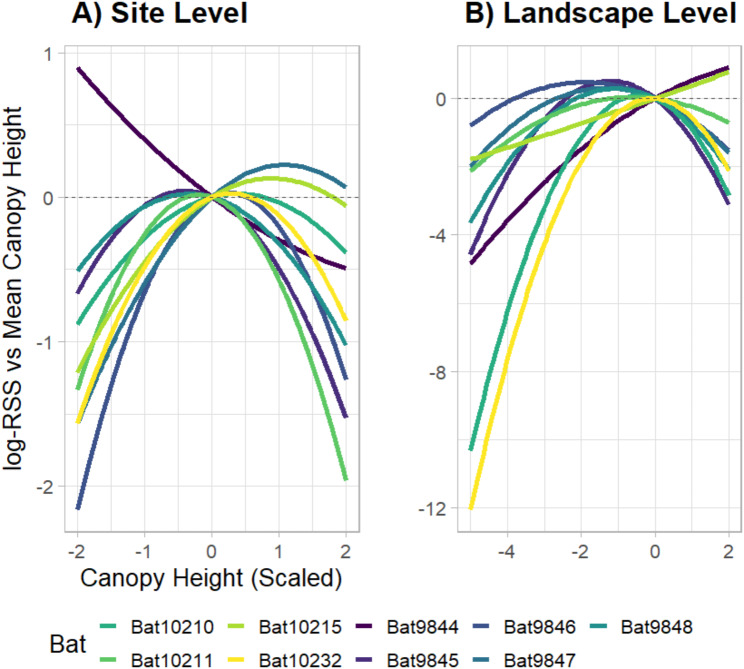
Log-transformed Relative Selection Strength (log-RSS) for each value of canopy height relative to the mean (indicated by x = 0) at the **(A)** site level (25 km^2^) and **(B)** landscape level (full movement trajectories). Each line represents an individual bat. Negative selection for a canopy height value relative to the mean is indicated where the line takes on values less than y = 0, and positive selection is indicated where the lines take on values greater than y = 0. Note that the plots were generated using a different model structure, and that the limits of both axes differ between the plots

The quadratic term for canopy height strongly predicted site-level habitat selection for seven of the nine bats, indicating a nonlinear relationship between canopy height and habitat selection. Examining the Relative Selection Strength across the range of scaled canopy height values revealed that these seven bats moved preferentially among habitats with intermediate canopy height, where the scaled range [-1, 1] represents 13.9 to 32.0 m (Fig. 2); that is, we detected a peak in preference near the mean canopy height encountered by these bats. This nonlinear relationship was also significant at the population level (GLMM: *p* < 0.001; Table 1).

**Table 1:** Estimated effects of covariates on bat habitat selection at the Bouamir Site extent (25 km^2^). SE = standard error; PVD = Plant Volume Density. All covariates are described in Table S1

Covariate	Estimate (SE)	*p*-Value
Canopy Height	0.093920 (0.076826)	0.221520
Canopy Height^2^	-0.250313 (0.045223)	3.11e-08***
Vertical Complexity Index	-0.024639 (0.058583)	0.674065
Leaf Area Index	-0.008522 (0.038223)	0.823574
Distance to gap 50 m^2^	-0.154816 (0.114585)	0.176664
Distance to gap 500 m^2^	-0.369714 (0.111727)	0.000936***
PVD 10–15 m	0.007432 (0.054390)	0.891307
PVD 15–20 m	0.019122 (0.039091)	0.624734
Swamp	0.210982 (0.141709)	0.136529
log (Step Length + 1):Swamp	0.178383 (0.048899)	0.000264 ***
log (Step Length + 1)	0.032994 (0.023883)	0.167122
cos (Turn Angle)	-0.552583 (0.131214)	2.65e-05 ***

The number of asterisks (*) after a coefficient estimate corresponds to significance at the level of 0.05 (*), 0.01 (**), and 0.001 (***), respectively

**Fig. 3:**
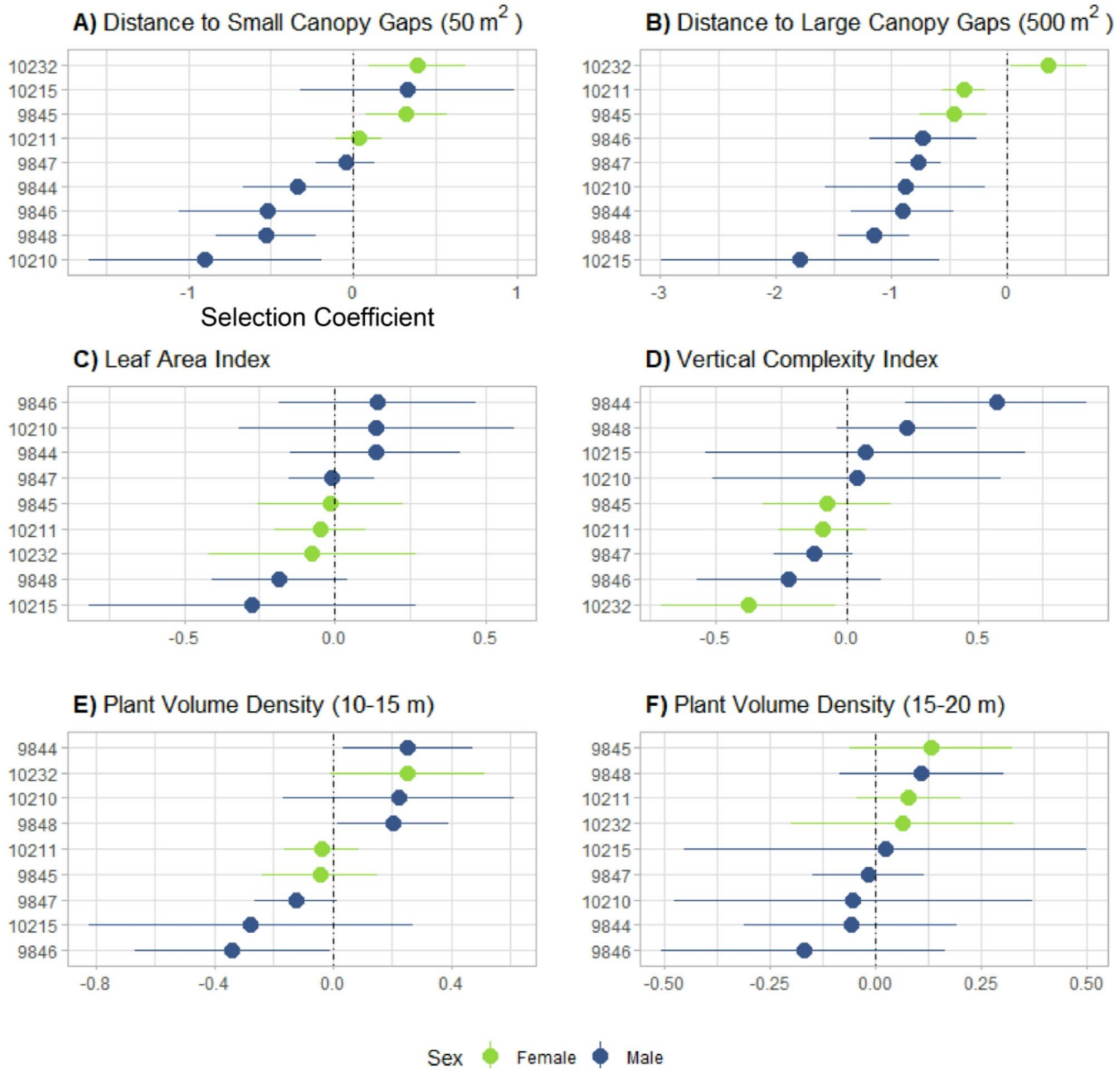
Selection coefficients and 95% confidence intervals (CIs) for each linear environmental predictor of bat movements within Bouamir Research Site (25 km^2^), including **(A)** Leaf Area Index, **(B)** Vertical Complexity Index, **(C)** Distance to small (50 m^2^ or greater) and **(D)** large (500 m^2^ or greater) canopy gaps, and **(E)** Plant Volume Density at a height of 10–15 and **(F)** 15–20 m. 95% CIs that do not overlap x = 0 indicate a significant effect of the covariate on individual bat habitat selection. Each bat is represented in the y-axes. Note that the order of bats differs for each plot

Three of the nine bats preferred areas closer to canopy gaps of at least 50 m^2^ (Fig. 3A), and eight bats preferred areas closer to large canopy gaps (500 m^2^ or larger; Fig. 3B). At the population level, hammer-headed fruit bats preferentially selected habitats closer to large canopy gaps (GLMM: *p* = 0.001; Table 1), but not small canopy gaps. We did not detect a significant influence of Leaf Area Index (Fig. 3C), Vertical Complexity Index (Fig. 3D), or Plant Volume Density at heights of 10–15 (Fig. 3E) or 15–20 m (Fig. 3F) on bat habitat selection at the population level (Table 1). Still, individual bats varied in their preference for these four structural attributes, displaying both positive and negative selection for vertical complexity and Plant Volume Density at heights of 10–15 m (Fig. 3). We also found that bats moved shorter distances in swamp habitats and were more likely to select other habitat types (*terra firma* forest and inselbergs) when moving longer distances (GLMM: *p* < 0.001; Table 1).

Used habitat calibration plots generally revealed an agreement between the distributions of each covariate in selected habitats and values predicted by the iSSA models fit to individual bats (Figs. S6-S16).

### Habitat selection at the landscape level

**Fig. 4:**
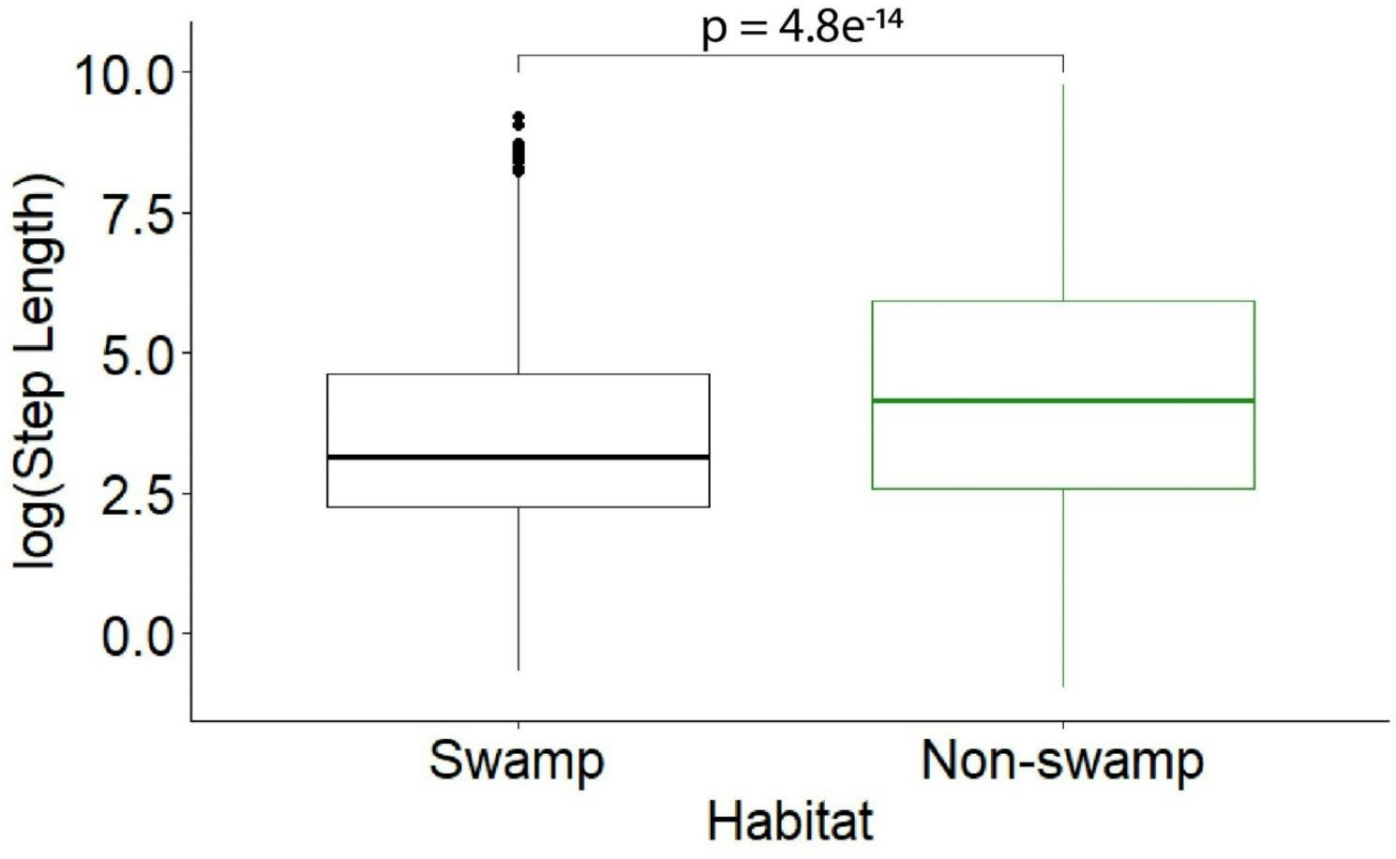
Difference in step length of bat movements (distance between successive GPS locations) between swamp and non-swamp habitats, including a Wilcoxon signed-rank test comparison (*p* = 4.8e^− 14^)

Landscape-level habitat selection refers to selection along the full extent of movement trajectories, including areas beyond the 25 km^2^ study site surveyed with UAV-LiDAR. At this spatial extent, hammer-headed bats selected for intermediate canopy height, and at a coarser spatial resolution (30 m) (GLMM: *p* < 0.001; Table 2; Fig. 2B). Bats also selected areas closer to canopy gaps at the coarser spatial resolution and greater extent (GLMM: *p* < 0.001; Table 2). At 100 m spatial resolution, bats selected for areas of greater canopy height heterogeneity (GLMM: *p* = 0.019; Table 2) but selected for lower canopy height heterogeneity at 1000 m resolution (GLMM: *p* < 0.001; Table 2). At the landscape extent, mean bat movement distances were 1.95 times greater through non-swamp habitats compared to swamps (GLMM: *p* < 0.001; Table 2; Wilcoxon signed-rank test: *p* = 4.8e^− 14^; Fig. 4).

**Table 2:** Estimated effects of covariates on bat habitat selection at the landscape extent. SE = standard error. All covariates are described in Table S1

Covariate	Estimate (SE)	*p*-Value
Canopy Height	-0.20391 (0.09409)	0.0302 *
Canopy Height^2^	-0.17474 (0.02818)	5.63e-10 ***
Distance to gap, threshold 15 m	-0.20490 (0.04563)	7.11e-06 ***
Canopy heterogeneity (100 m)	0.14199 (0.06948)	0.0410*
Canopy heterogeneity (1000 m)	-0.30371 (0.07040)	1.60e-05 ***
Swamp	-0.02541 (0.11487)	0.8250
log (Step Length + 1):Swamp	0.17861 (0.04233)	2.45e-05 ***
log (Step Length + 1)	-0.02517 (0.01665)	0.1307
cos (Turn Angle)	-0.63586 (0.11222)	1.46e-08 ***

The number of asterisks (*) after a coefficient estimate corresponds to significance at the level of 0.05 (*), 0.01 (**), and 0.001 (***), respectively

Used habitat calibration plots applied to landscape-level iSSAs generally yielded narrower simulation envelopes than those produced by site-level iSSAs, and exhibited agreement between the distributions of each covariate in selected habitats and values predicted by the models (Figs. S17-S23). These findings indicate that the iSSA models were well-calibrated with an appropriate set of predictors [39].

### Movement behavior

**Fig. 5:**
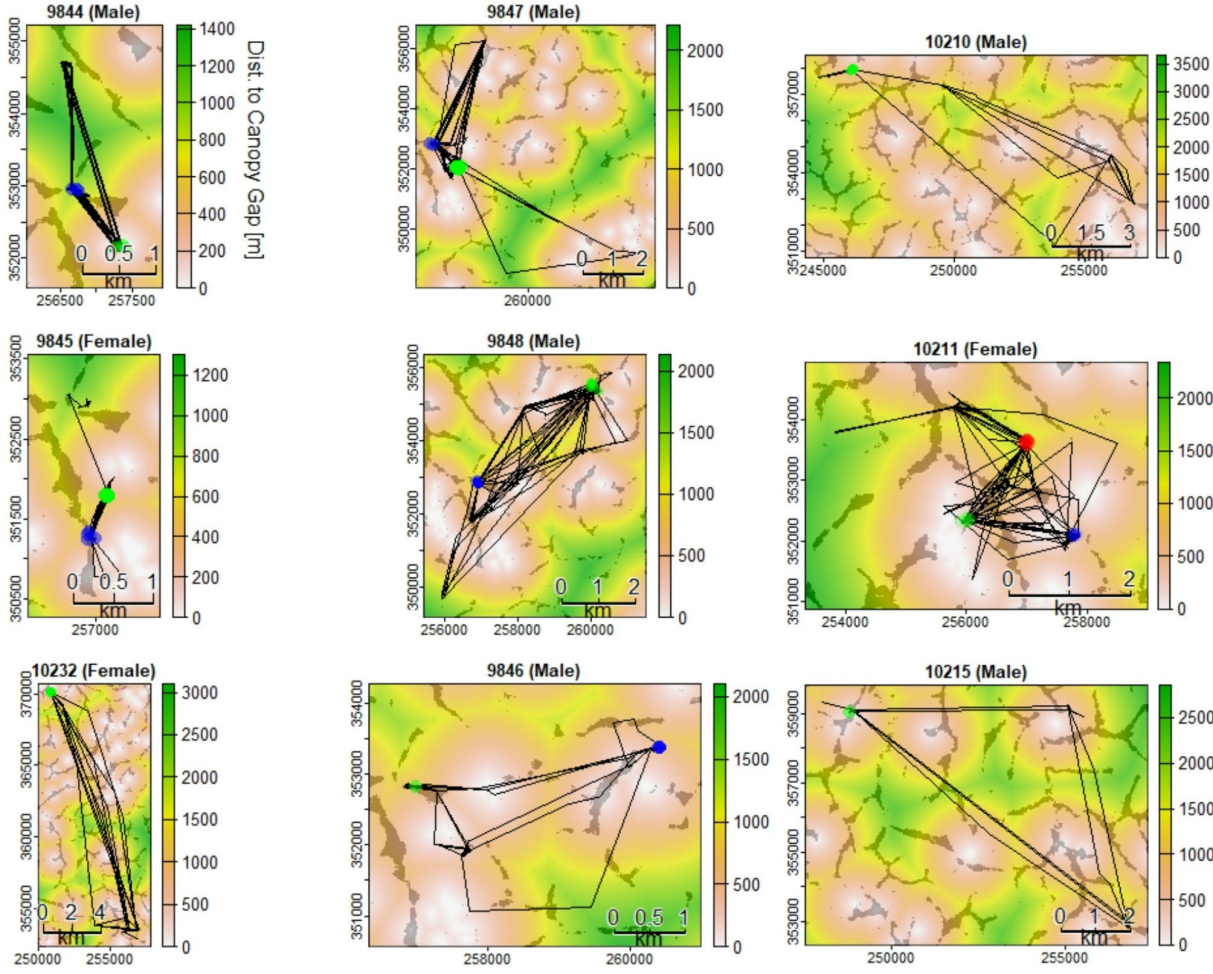
Movement trajectories of each bat plotted over distance to canopy gap (15 m threshold) and the distribution of swamp habitats (gray polygons). Clusters of green, blue, and red points represent areas with the greatest revisitation rates (75th percentile or greater)

For each bat, we used *k*-means clustering to identify 1–3 sites with recursive movements in the 75th percentile (Fig. 5), indicating locations of high probability of use. Recursive movements to high-use locations varied among bats but ranged from eight to 63 visits. All bats displaced at least 3 km from their capture location (Fig. S3). In a single night, bats flew total distances up to 42.3 km (mean ± SD: 10.8 ± 10.2 m). The greatest distance a bat displaced from the tagging location was 18.3 km (Fig. 5); during this flight, a female bat (ID:10232) flew 17.7 km within 30 min (Fig. S4) and left the protected Dja Faunal Reserve to enter a human-settled landscape (Fig. 1B). We detected a nonlinear relationship between step lengths and hours after sunset (GAMM: R^2^ = 0.067; *p* < 0.001), with many individuals moving the greatest distances shortly after sunset and again before sunrise (Fig. S5).

## Discussion

In this study, we showed how hammer-headed bats select habitats with respect to 3D vegetation structure at the landscape scale. At the population level, bats preferred areas of intermediate canopy height and areas close to large canopy gaps. This relationship was evident at both site-level (25 km^2^) and landscape-level (i.e., entire movement trajectory) extents. However, individual variation in selection for other features of 3D vegetation structure—including vertical complexity, Plant Volume Density, and distance to smaller canopy gaps—indicated that variation in both vertical and horizontal vegetation structure is important for supporting a population’s foraging and roosting behaviors.

Bats moved shorter distances in swamps compared to other habitat types. Movement distances can indicate resource tracking behavior; for example, large birds generally move longer distances through homogeneous habitats to meet resource needs [58]. Black-casqued hornbills (*Ceratogymna atrata*) exhibit a similar behavior to bats at the Bouamir Research Site, selecting swamps during hotter temperatures and becoming less active [59]; swamps dominated by *Raphia* palms likely provide a cool location for a day roost and dense vegetation that may conceal birds and bats from predators. Still, we did not detect a population-level signal of bat selection for Plant Volume Density of mid-story vegetation strata (10–20 m), albeit with a small sample size and two-dimensional tracking methods. Swamp habitats occur throughout Cameroon’s rainforest zone and may be a necessary landscape feature for hammer-headed bat populations. Indeed, hammer-headed bats in the Republic of the Congo preferred areas near watercourses, which could also indicate a preference for foraging or roosting in wetlands [27]. In addition, figs (*Ficus* spp.) are an important component of fruit bat diets that occur frequently along Central African waterways [60].

The preference of several individuals to move among areas close to large canopy gaps may also reflect selection of trees that produce abundant fruits with small seeds. *Musanga cecropioides* is one of the preferred species in the hammer-headed bat’s diet [27], and it typically grows in disturbed and early successional areas, with some mature trees persisting in mature rainforest [61]. Although we did not determine which other species might be consumed by hammer-headed bats in the area, small-seeded tree species like *M. cecropioides* are typically efficient colonizers of open, disturbed habitats [62] and large natural canopy gaps. Our results show that individual bats vary widely in their preference for other attributes of 3D vegetation structure, which may influence their roles as seed dispersers. Individual animals exhibit movement “personalities”, or behavioral types, that reflect different preferences in space use and have been hypothesized to influence spatial patterns of seed dispersal [63–65]. Such individual variation indicates the importance of landscape heterogeneity in supporting animal populations and their ecological roles, which can influence landscape heterogeneity in turn through seed dispersal [26]. Although fragmentation can limit many large-bodied frugivores from dispersing seeds among forest patches, smaller frugivores such as bats may play a key role in reforestation and recovery of aboveground carbon stocks by frequenting villages and canopy gaps [66].

Canopy height heterogeneity is a measurement of horizontal complexity in vegetation structure. At the landscape extent, bats selected areas of greater heterogeneity at the 100 m scale. Swamps, inselbergs, and the research camp where all bats were captured typically had high height heterogeneity. At the 1000 m scale, horizontal variation in vegetation structure was high in a region to the south of where bats were captured, marked by a high concentration of inselbergs. Accordingly, the 1000 m scale may not be relevant to the scale of home range selection by many of the bats. The positive population-level selection for canopy height heterogeneity may reflect a preference for transitional areas between forest and swamp or forest and inselberg.

Very few studies report animal movement data from the Central African tropics, and these studies are mainly from a limited number of taxa and intensively studied locations [67, 68]. Compounding this issue is the difficulty of tracking bats over multiple seasons due to limitations in battery life of tags [69]. Hammer-headed bats are thought to migrate long distances, but tracking technology has not yet revealed the nature of these events. During the short period we tracked hammer-headed bats (3–15 nights), we recorded displacements up to 17.7 km from roosting locations in a single night. These distances were greater than those reported from other studies of this species over a similar time period, but unlike these previous studies [27, 29], individuals in our study were not tagged at leks. Future tracking studies that capture seasonal variation in hammer-headed bat movement, including migrations, will be invaluable for characterizing this species’ behaviors and their consequences for ecosystem functioning and disease transmission.

Nightly movements of hammer-headed bats were relatively predictable, with repeated visits, or recursions, to one to three locations over the duration of the tracking period. Although these locations were sometimes several kilometers apart, bats frequently exhibited directed movements, with long movement steps and turn angles near zero. Bats typically undertook these long, directed flights shortly after leaving the roost at sunset. These observations provide further evidence of fruit bats’ advanced spatial memory. The Egyptian fruit bat, a related species, has been shown to possess a “cognitive map” of roosts and fruiting trees and develop shortcuts among these locations in areas with more open vegetation structure [25]. In more complex environments, such as tropical rainforests, spatial memory is thought to be less useful for animal movement due to the costs of processing information [70]. Hammer-headed bats might overcome this problem by moving among easily distinguishable landscape features, such as inselbergs, which create large canopy gaps. Longer-term tracking studies would reveal how bats navigate and find new resources when fruits are depleted at repeatedly visited trees.

Although long-term studies of fruit bat movements are still challenging due to tradeoffs in tracking technology, an important step towards understanding seasonal variation in fruit bat habitat selection is to characterize both vertical and horizontal vegetation structure at spatial extents that cover the full range of their movements. We addressed this challenge by upscaling canopy height, gap, and heterogeneity metrics from a 25 km^2^ UAV-LiDAR study area to a 494,000 km^2^ study area covering most of southern Cameroon and neighboring regions. Hammer-headed fruit bats forage in open spaces, roost in dense vegetation, and commute long distances across landscapes containing forests, wetlands, inselbergs, waterways, villages, agriculture, and other anthropogenic features [27]. NASA’s GEDI mission enabled us to characterize 3D structure at a broader extent than what is possible with UAV-LiDAR alone [14]. Still, UAV-LiDAR surveys characterize 3D vegetation structure at much higher spatial resolution [71], so it is advantageous to investigate habitat selection by integrating UAV-LiDAR and spaceborne LiDAR, as we showed in this study. We expect this approach to be applicable to any study system in temperate and tropical regions where GEDI measurements are available.

## Conclusions

Tropical forests are hotspots for biodiversity, due in part to their high structural complexity [21]. Tropical humid forests exhibit high structural complexity in both vertical and horizontal dimensions, and our study showed that hammer-headed bats require a wide variety of vegetation cover types, including open space near canopy gaps, swamp habitats, and forests of intermediate height. In human-settled areas, hammer-headed bats move primarily among agricultural areas and waterways, likely driven by the need to find fruits [27]. Understanding how hammer-headed bats move among foraging and roosting sites in mature rainforest habitat can lend insight into the habitat requirements necessary to promote their role as seed dispersers and limit the risk of viral spillover events. Integrating remote sensing methods to produce metrics relevant to animal habitat selection is an important step towards linking landscape patterns to ecological processes.

## Electronic supplementary material

Below is the link to the electronic supplementary material.

Supplementary Material 1

## Data Availability

The data and code supporting this study’s findings are available in Dryad: (10.5061/dryad.7m0cfxq4t). Bat GPS locations are available by request on Movebank (movebank.org, study name “Hypsignathus monstrosus, Dja Reserve,” study ID 2988162659).
