# Supplementary material for: Spaceborne and UAV-LiDAR reveal hammer-headed bat preference for intermediate canopy height and diverse structure in a Central African rainforest
Source: Mov Ecol. 2025 Apr 22;13:30. doi: 10.1186/s40462-025-00552-7 (PMC12016133; doi:10.1186/s40462-025-00552-7)
Supplement: Supplementary file 1 — Supplementary Material 1 [file 40462_2025_552_MOESM1_ESM.docx]

**Supplemental material**

**Table S1:** Descriptions of covariates included in model selection for integrated Step Selection Analyses at two different spatial extents: “Bouamir Site” (25 km^2^) and “Landscape” (Full bat trajectories).

| **Covariate** | **Definition** | **Spatial resolution** | **Spatial Extent** |
| --- | --- | --- | --- |
| Canopy Height | Height of first lidar return | 10 m | Bouamir Site |
| Canopy Height (Upscaled) | Height of 95^th^ percentile of first lidar returns, with gaps interpolated using Machine Learning | 30 m | Landscape |
| Vertical Complexity Index | A fixed normalization of the entropy function, based on the 3D point cloud | 10 m | Bouamir Site |
| Leaf Area Index | Density of vegetation material, calculated from the 3D point cloud as the sum of leaf area density profiles at 5 m intervals throughout the vertical column | 10 m | Bouamir Site |
| Distance to gap ≥50 m^2^ | Distance to gap of minimum size 50 m^2^, 5 m height threshold | 10 m | Bouamir Site |
| Distance to gap ≥500 m^2^ | Distance to gap of minimum size 500 m^2^, 5 m height threshold | 10 m | Bouamir Site |
| Distance to gap, 15 m threshold | Distance to canopy gaps of 15 m threshold, and with area not exceeding 10,000 m^2^ | 30 m | Landscape |
| Plant Volume Density (10-15 m) | Volume of plant area (wood and foliage) 10-15 m above the ground | 10 m | Bouamir Site |
| Plant Volume Density (15-20 m) | Volume of plant area (wood and foliage) 15-20 m above the ground | 10 m | Bouamir Site |
| Canopy Heterogeneity (100m) | Standard deviation of “Canopy Height Upscaled”, aggregated to 100 m | 100 m | Landscape |
| Canopy Heterogeneity (1000m) | Standard deviation of “Canopy Height Upscaled”, aggregated to 1000 m | 1000 m | Landscape |
| Swamp | Habitat class defined as swamp, all other classes (*terra firme* forest, inselberg) taking on zero | 10 m | Bouamir Site/  Landscape |
| Step length | Distance between two successive GPS locations | NA | Bouamir Site/  Landscape |
| Turn angle | Angle between two successive GPS locations | NA | Bouamir Site/  Landscape |
| Step length:Swamp | Interaction between step length and use of habitat class defined as swamp, all other classes (*terra firme* forest, inselberg) taking on zero | 10 m | Bouamir Site/  Landscape |
| Step ID | Stratum consisting of a selected step (n=1) and randomly generated steps (n=10) | NA | Bouamir Site/  Landscape |


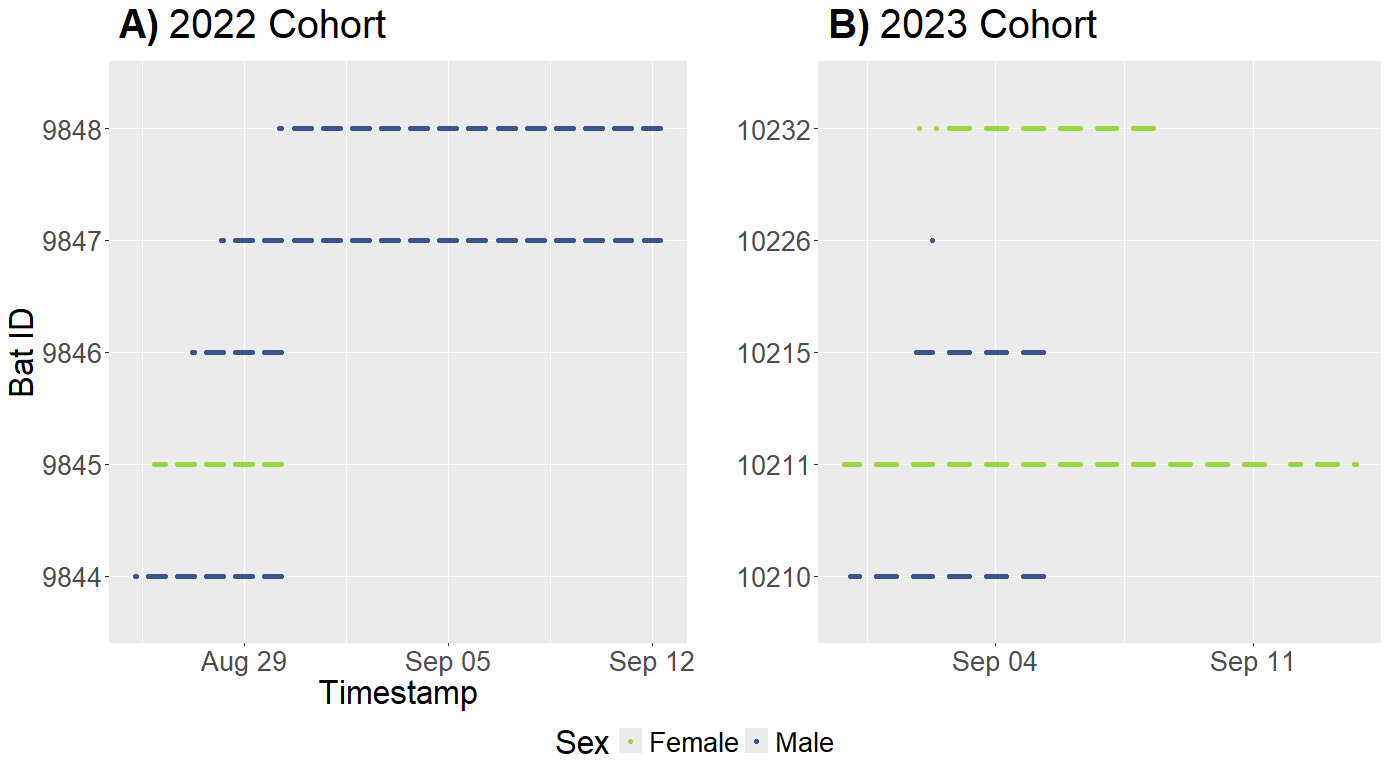


**Figure S1:** Sample periods of bats tracked in **A)** 2022 and **B)** 2023.

**
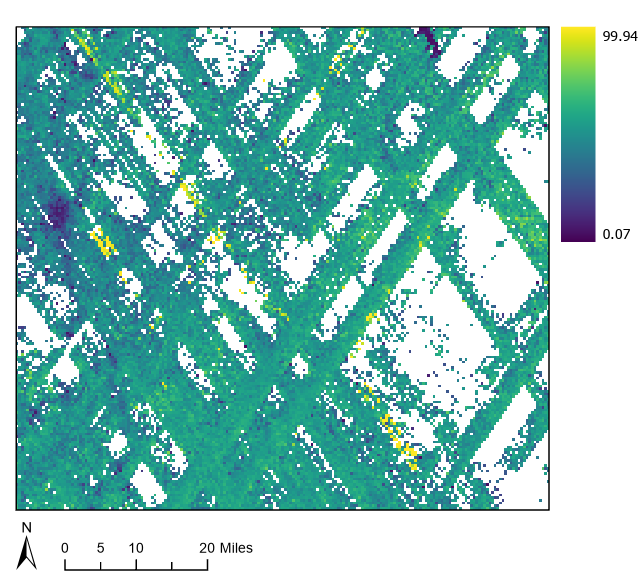
**

**Figure S2:** GEDI flag-filtered data aggregated by median and colored by canopy height value. The bright yellow patterning indicates erroneous data along an orbital track—canopy height values in Congo Basin forests should not reach nearly 100 m.

**
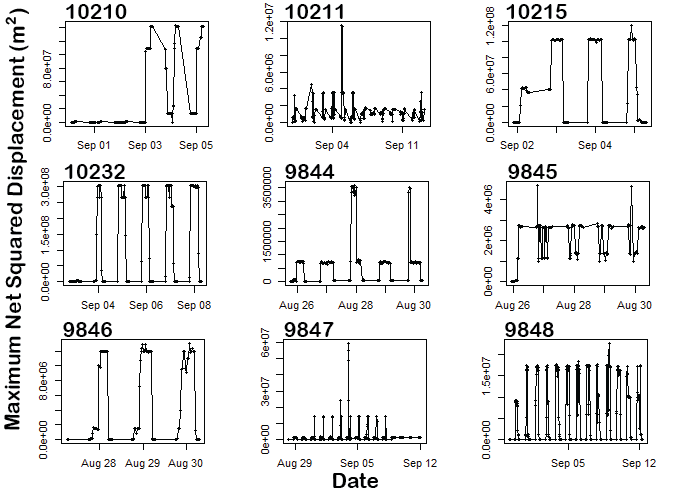
**

**Figure S3:** Maximum Net Squared Displacement of each bat throughout its tracking period relative to the starting location, which in most cases (except ID:9845) was near the bat’s regular roosting location.

**
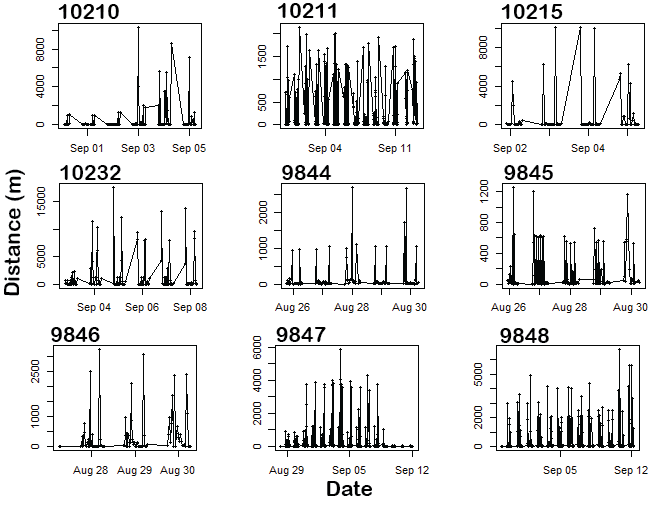
**

**Figure S4:** Distances between successive relocations for the full tracking period of all bats (n=9), with a constant sampling interval of 30 mins.


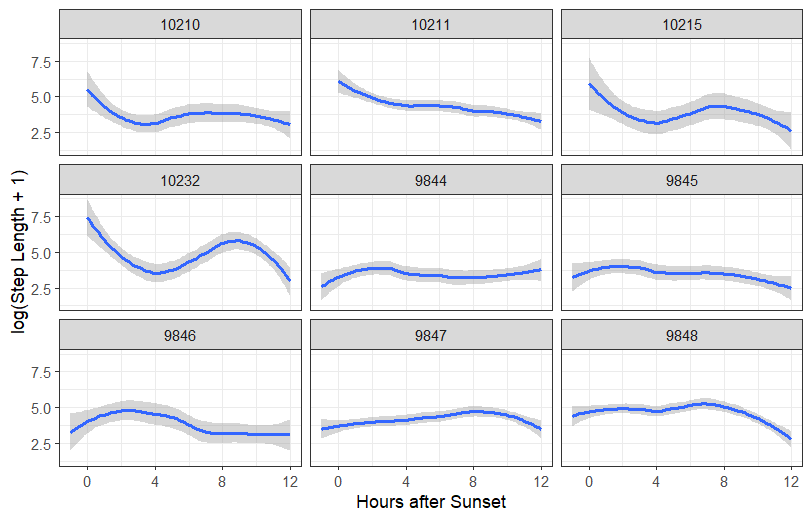


**Figure S5:** Smoothed relationship between step lengths (log transformed) and hours after sunset for each individual bat (n=9). The gray shaded area represents the 95% Confidence Interval.


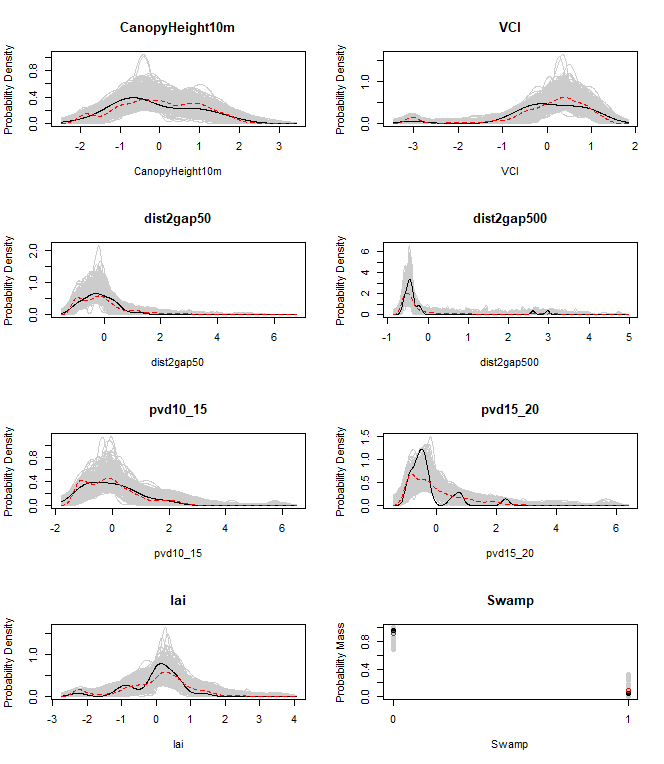


**Figure S6:** Site-level used habitat calibration (UHC) plot for bat 9844. Black lines represent the distribution of the covariate in selected habitat, red dotted lines represent the distribution of the covariate in available habitat, and grey lines represent 1000 simulations based on the site-level iSSA model.


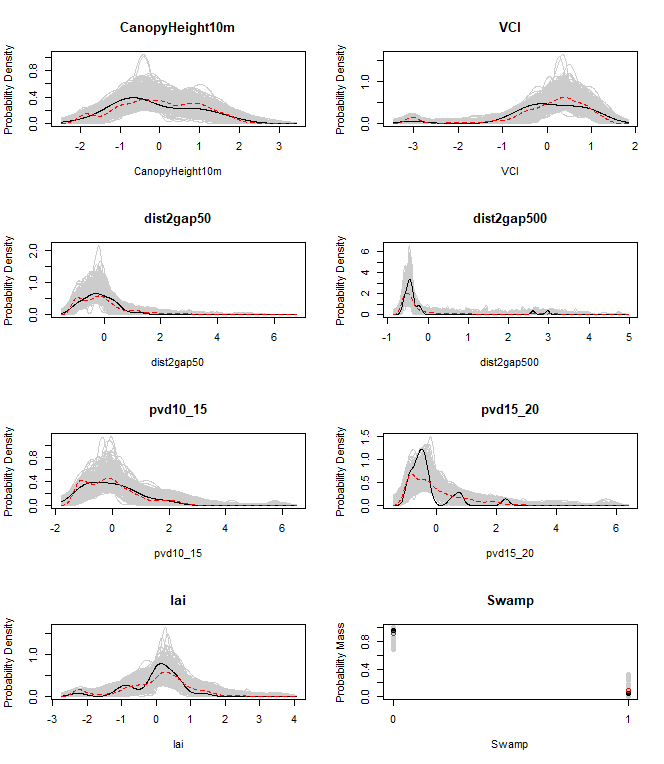


**Figure S7:** Site-level used habitat calibration (UHC) plot for bat 9845. Black lines represent the distribution of the covariate in selected habitat, red dotted lines represent the distribution of the covariate in available habitat, and grey lines represent 1000 simulations based on the site-level iSSA model.


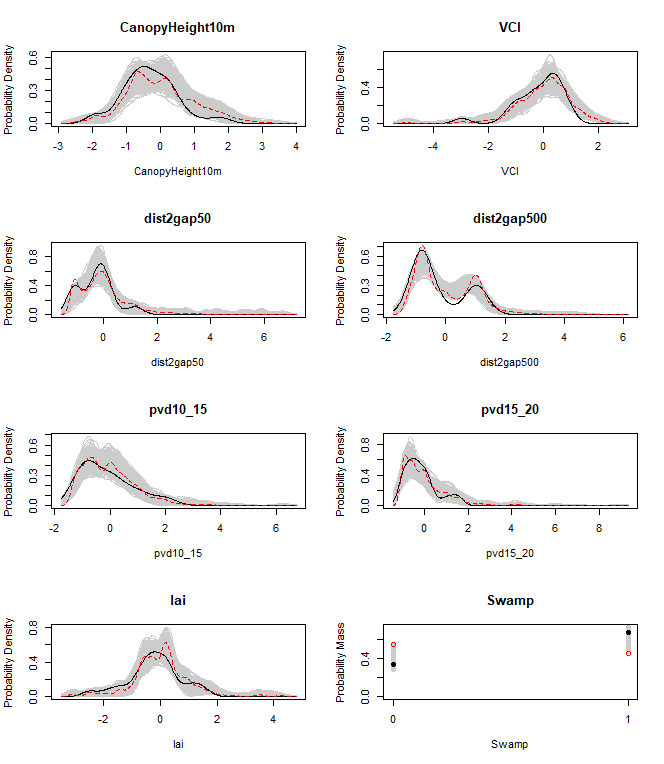


**Figure S8:** Site-level used habitat calibration (UHC) plot for bat 9846. Black lines represent the distribution of the covariate in selected habitat, red dotted lines represent the distribution of the covariate in available habitat, and grey lines represent 1000 simulations based on the site-level iSSA model.


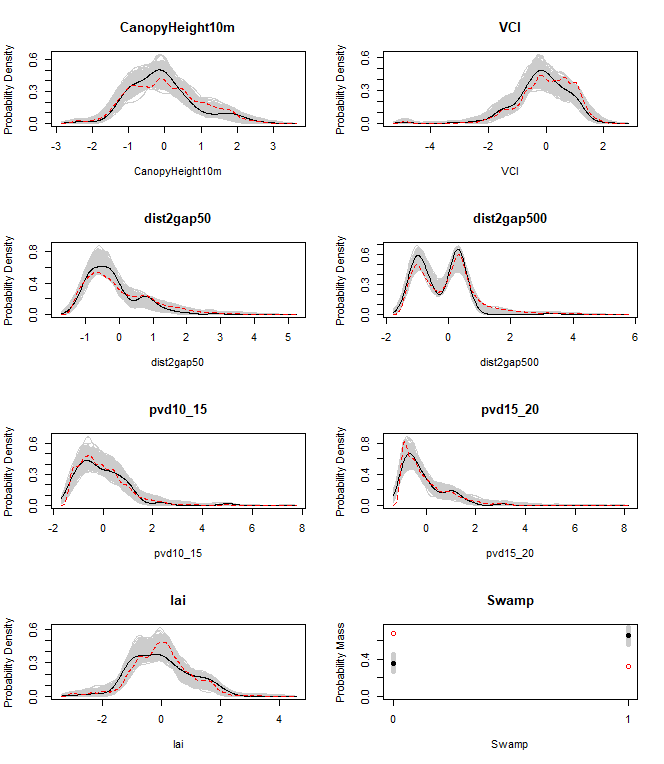


**Figure S9:** Site-level used habitat calibration (UHC) plot for bat 9847. Black lines represent the distribution of the covariate in selected habitat, red dotted lines represent the distribution of the covariate in available habitat, and grey lines represent 1000 simulations based on the site-level iSSA model.


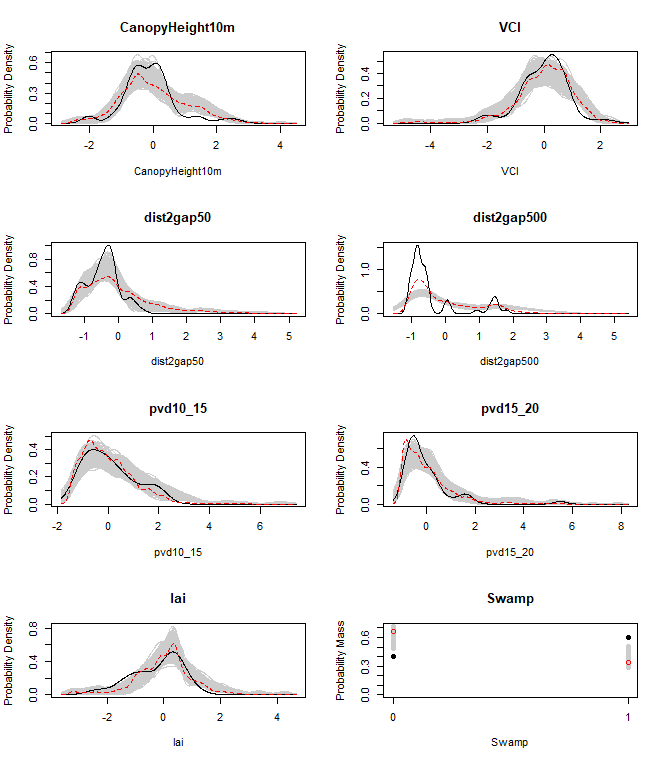


**Figure S10:** Site-level used habitat calibration (UHC) plot for bat 9848. Black lines represent the distribution of the covariate in selected habitat, red dotted lines represent the distribution of the covariate in available habitat, and grey lines represent 1000 simulations based on the site-level iSSA model.

**
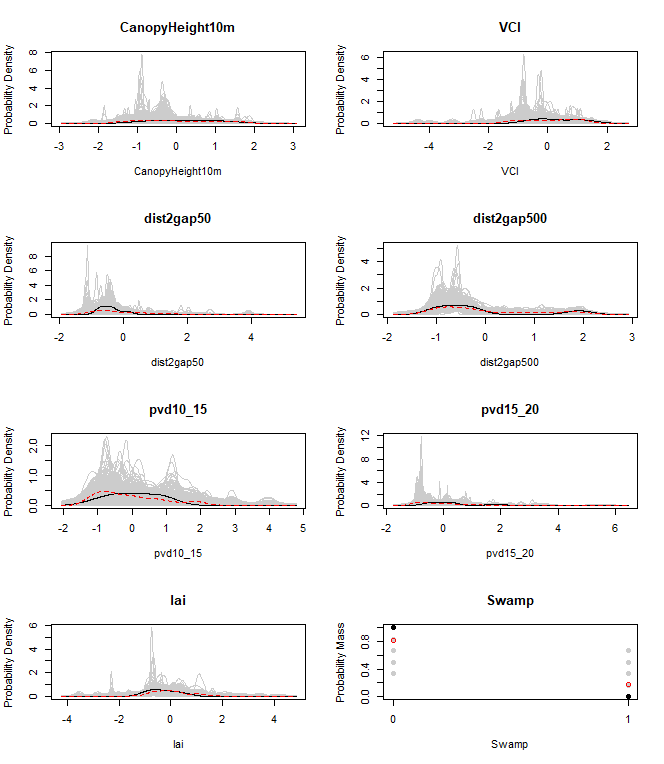
**

**Figure S11:** Site-level used habitat calibration (UHC) plot for bat 10210. Black lines represent the distribution of the covariate in selected habitat, red dotted lines represent the distribution of the covariate in available habitat, and grey lines represent 1000 simulations based on the site-level iSSA model.


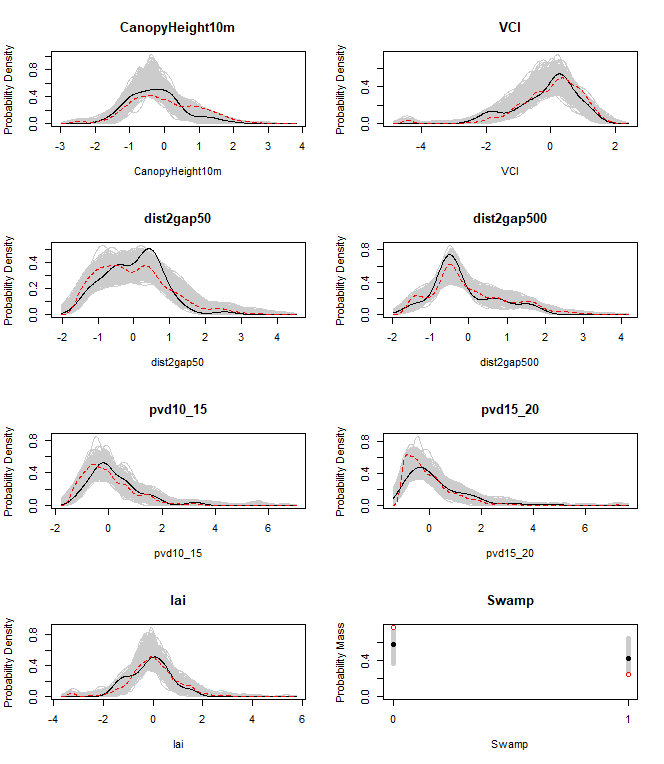


**Figure S12:** Site-level used habitat calibration (UHC) plot for bat 10211. Black lines represent the distribution of the covariate in selected habitat, red dotted lines represent the distribution of the covariate in available habitat, and grey lines represent 1000 simulations based on the site-level iSSA model.


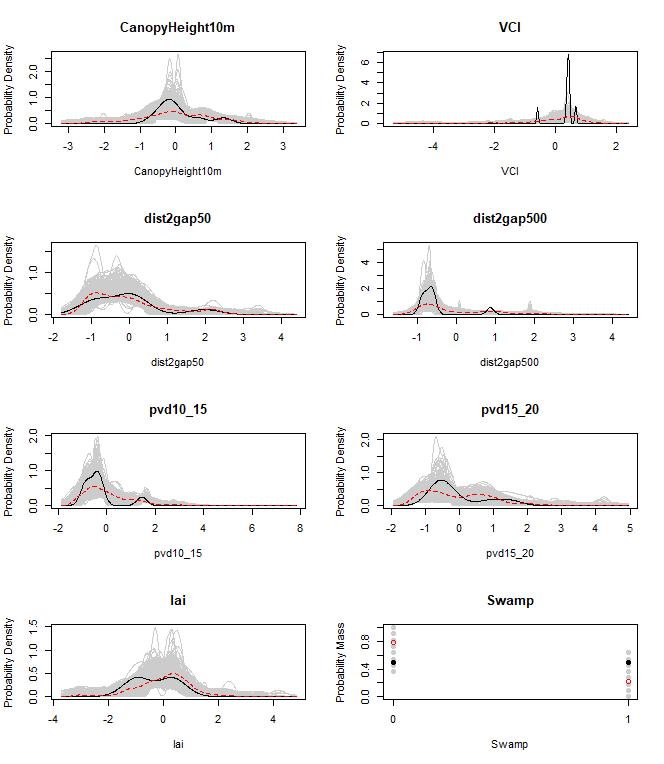


**Figure S13:** Site-level used habitat calibration (UHC) plot for bat 10215. Black lines represent the distribution of the covariate in selected habitat, red dotted lines represent the distribution of the covariate in available habitat, and grey lines represent 1000 simulations based on the site-level iSSA model.


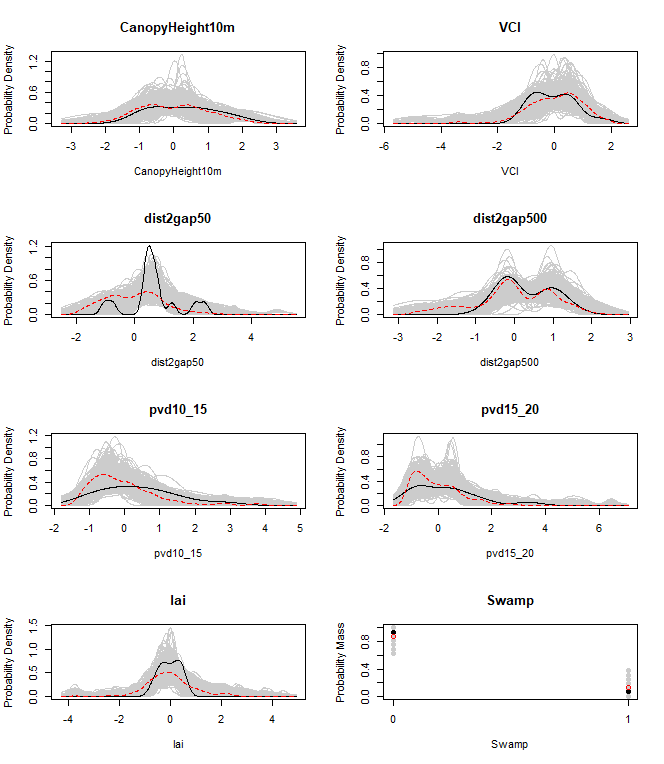


**Figure S14:** Site-level used habitat calibration (UHC) plot for bat 10232. Black lines represent the distribution of the covariate in selected habitat, red dotted lines represent the distribution of the covariate in available habitat, and grey lines represent 1000 simulations based on the site-level iSSA model.


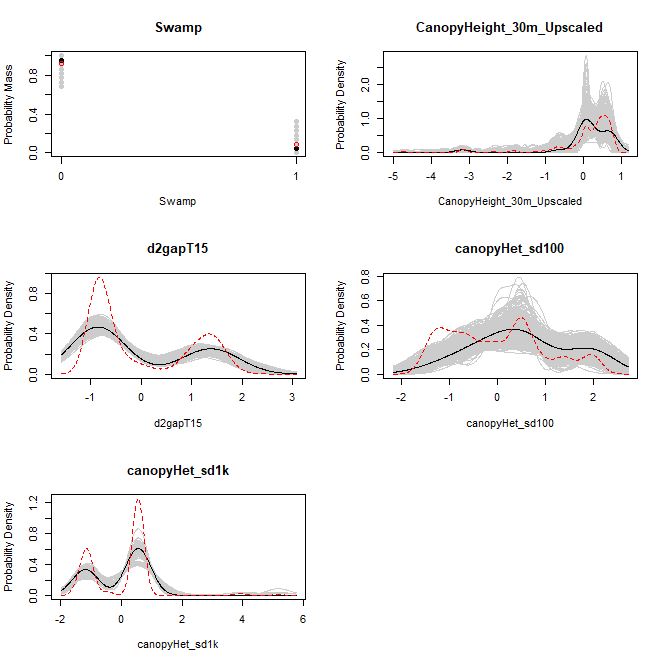


**Figure S15:** Landscape-level used habitat calibration (UHC) plot for bat 9844. Black lines represent the distribution of the covariate in selected habitat, red dotted lines represent the distribution of the covariate in available habitat, and grey lines represent 1000 simulations based on the landscape-level iSSA model.


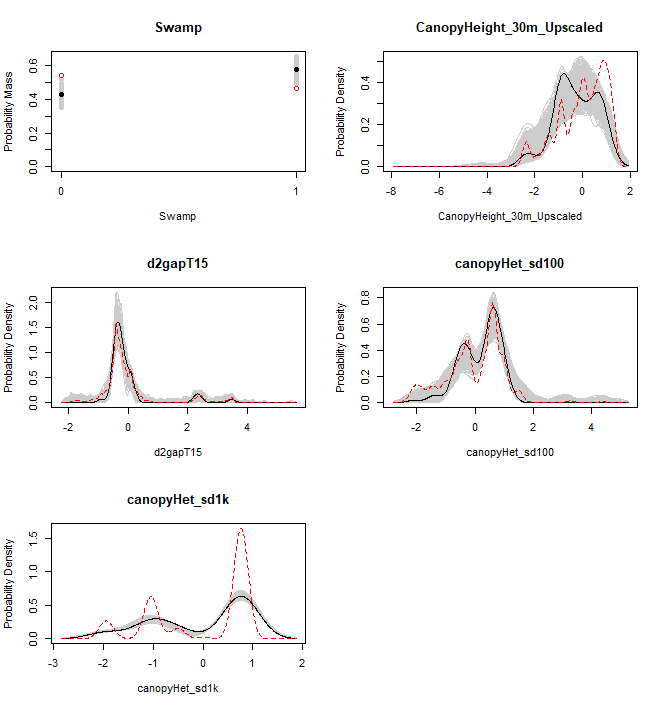


**Figure S16:** Landscape-level used habitat calibration (UHC) plot for bat 9845. Black lines represent the distribution of the covariate in selected habitat, red dotted lines represent the distribution of the covariate in available habitat, and grey lines represent 1000 simulations based on the landscape-level iSSA model.


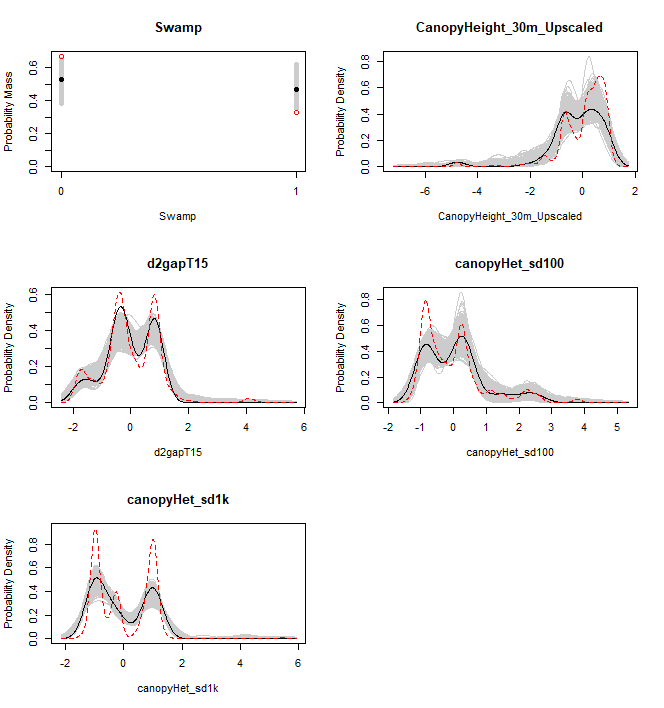


**Figure S17:** Landscape-level used habitat calibration (UHC) plot for bat 9846. Black lines represent the distribution of the covariate in selected habitat, red dotted lines represent the distribution of the covariate in available habitat, and grey lines represent 1000 simulations based on the landscape-level iSSA model.


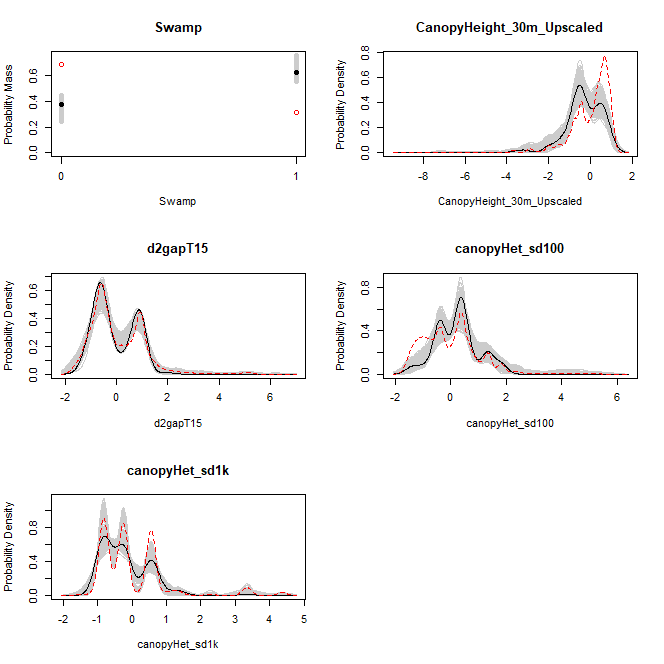


**Figure S18:** Landscape-level used habitat calibration (UHC) plot for bat 9847. Black lines represent the distribution of the covariate in selected habitat, red dotted lines represent the distribution of the covariate in available habitat, and grey lines represent 1000 simulations based on the landscape-level iSSA model.


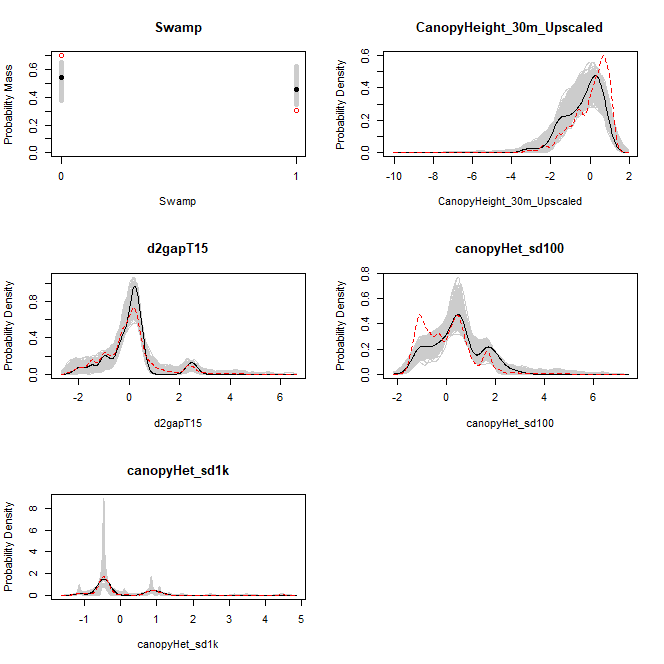


**Figure S19:** Landscape-level used habitat calibration (UHC) plot for bat 9848. Black lines represent the distribution of the covariate in selected habitat, red dotted lines represent the distribution of the covariate in available habitat, and grey lines represent 1000 simulations based on the landscape-level iSSA model.


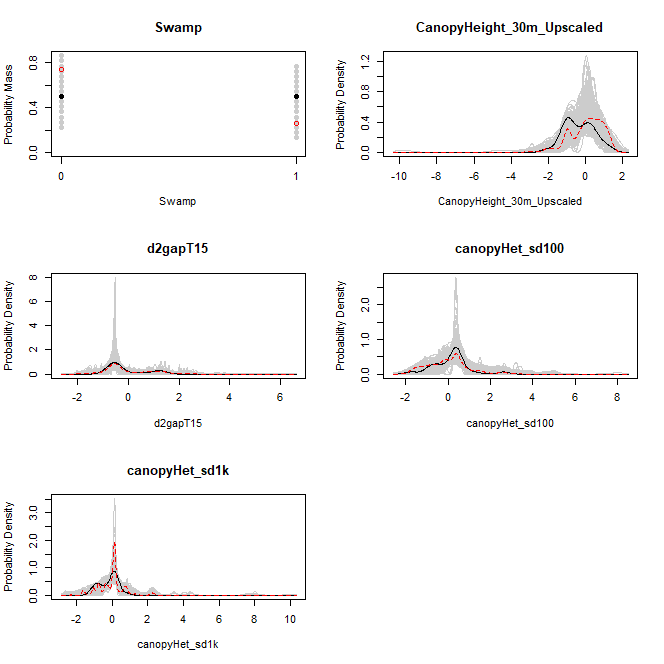


**Figure S20:** Landscape-level used habitat calibration (UHC) plot for bat 10210. Black lines represent the distribution of the covariate in selected habitat, red dotted lines represent the distribution of the covariate in available habitat, and grey lines represent 1000 simulations based on the landscape-level iSSA model.


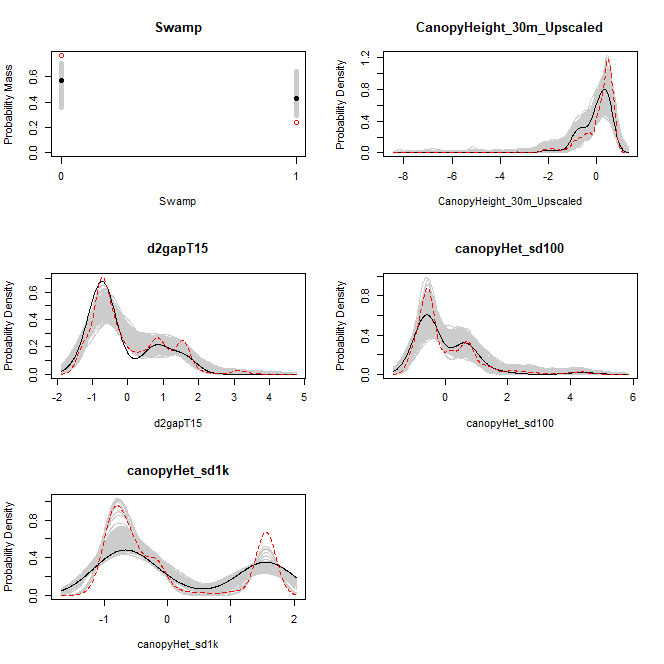


**Figure S21:** Landscape-level used habitat calibration (UHC) plot for bat 10211. Black lines represent the distribution of the covariate in selected habitat, red dotted lines represent the distribution of the covariate in available habitat, and grey lines represent 1000 simulations based on the landscape-level iSSA model.


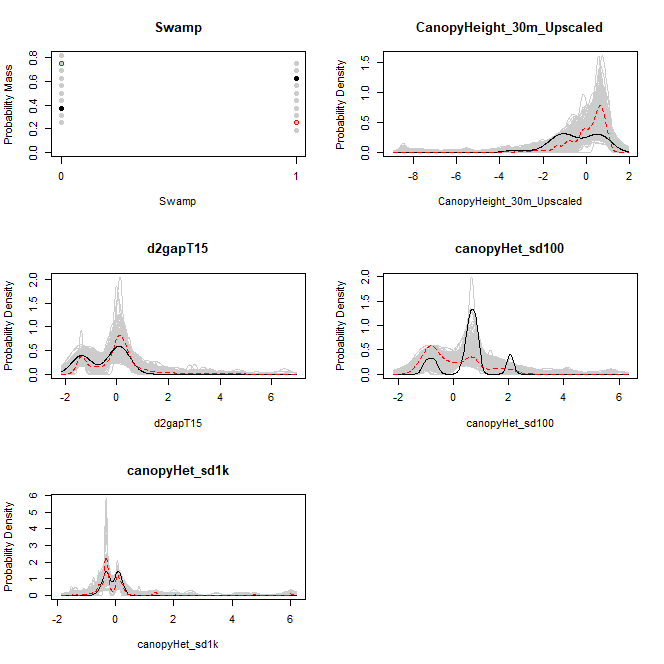


**Figure S22:** Landscape-level used habitat calibration (UHC) plot for bat 10215. Black lines represent the distribution of the covariate in selected habitat, red dotted lines represent the distribution of the covariate in available habitat, and grey lines represent 1000 simulations based on the landscape-level iSSA model.


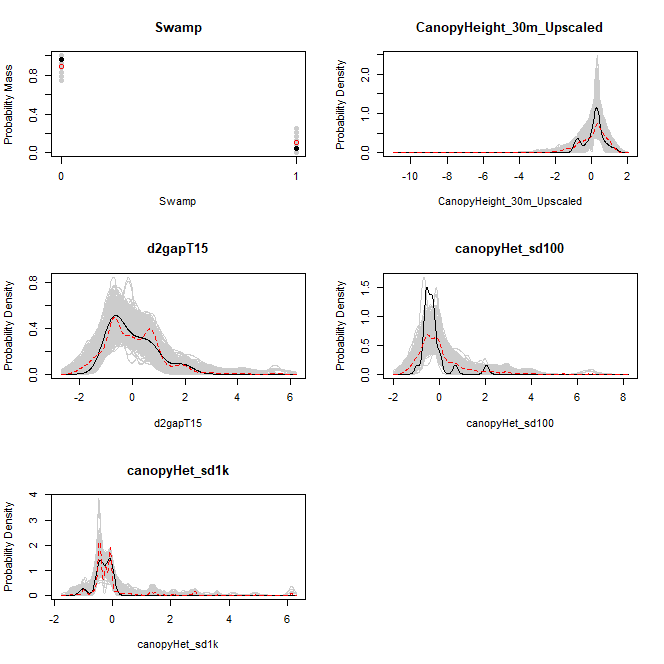


**Figure S23:** Landscape-level used habitat calibration (UHC) plot for bat 10232. Black lines represent the distribution of the covariate in selected habitat, red dotted lines represent the distribution of the covariate in available habitat, and grey lines represent 1000 simulations based on the landscape-level iSSA model.
